# 2,2′,4,4′-Tetrabromodiphenyl Ether (BDE-47) at Environmental Levels Influenced Photosynthesis in the Mangrove Species *Kandelia obovata*

**DOI:** 10.3390/toxics12070456

**Published:** 2024-06-25

**Authors:** Meijing Xue, Yajun Shi, Jing Xiang, Yan Zhang, Hanxun Qiu, Wenming Chen, Jiliang Zhang

**Affiliations:** 1 Ministry of Education Key Laboratory for Ecology of Tropical Islands, Key Laboratory of Tropical Animal and Plant Ecology of Hainan Province, College of Life Sciences, Hainan Normal University, Haikou 571158, China; 18404983033@163.com (M.X.); 202212071300014@hainnu.edu.cn (Y.S.); xiangj2018@163.com (J.X.); 202311071300008@hainnu.edu.cn (Y.Z.); qiuhxun@163.com (H.Q.); wenmingchen@aliyun.com (W.C.); 2 Hainan Provincial Key Laboratory of Ecological Civilization and Integrated Land-Sea Development, Hainan Normal University, Haikou 571158, China

**Keywords:** BDE-47, *Kandelia obovata*, photosynthesis, transcriptome, photosystem

## Abstract

2,2′,4,4′-tetra-bromodiphenytol ether (BDE-47) is one of the ubiquitous organic pollutants in mangrove sediments. To reveal the toxic effects of BDE-47 on mangrove plants, the mangrove species *Kandelia obovate* was used to investigate the photosynthetic capacity effects and the molecular mechanisms involved after BDE-47 exposure at environment-related levels (50, 500, and 5000 ng g^−1^ dw). After a 60-day exposure, the photosynthetic capacity was inhibited in *K. obovata* seedlings, and a decrease in the stomatal density and damage in the chloroplast ultrastructure in the leaves were found. Transcriptome sequencing showed that, following exposure to BDE-47, gene expression in photosynthesis-related pathways was predominantly suppressed in the leaves. The bioinformatics analysis indicated that BDE-47 exerts toxicity by inhibiting photosystem I activity and chlorophyll a/b-binding protein-related genes in the leaves of *K. obovata*. Thus, this study provides preliminary theoretical evidence for the toxic mechanism effect of BDE-47 on photosynthesis in mangrove species.

## 1. Introduction

The mangrove forest located in the coastal intertidal wetland of tropical and subtropical regions is a unique and vital coastal ecosystem that connects the land and sea [1]. The 2020 mangrove area statistics revealed that the global mangrove coverage spanned a total area of 145,068 square kilometers, with Asia boasting the largest proportion at 39.2% [2]. Being one of the most carbon-rich ecosystems globally, mangrove forests exhibit a stronger carbon capacity compared to any other terrestrial ecosystem [3]. The total carbon stock of mangroves exceeds 450.6 Mg C ha^−1^ [4], thus playing a distinctive role in reducing the levels of carbon dioxide and greenhouse gases in the atmosphere [5]. Mangroves not only provide crucial habitats, spawning ground, and food sources for marine and terrestrial organisms but also play a significant role in mitigating coastal erosion while resisting storm surges and tsunamis [1,6]. However, due to their location at the interface of land, freshwater, and ocean, mangroves are highly susceptible to disruption resulting from human activities, such as pollution inputs including overfishing, exploitation, municipal household waste disposal practices, domestic wastewater discharge along with aquaculture operations and mariculture activities, shipping-related impacts as well as shore-based industries [7]. The increasing threats faced by mangrove forests led to their area declining worldwide. In fact, during the 1980s and 1990s, approximately 35% of the global mangrove areas were lost, while nearly two-thirds of mangrove forests in China have disappeared [8].

Sediments in the mangrove ecosystem serve as important media for the filtration and adsorption of pollutants in the intertidal zone between land and sea [9,10]. These sediments serve as both the fundamental support for the survival of mangroves and the primary reservoir of persistent organic pollutants (POPs), such as polybrominated diphenyl ethers (PBDEs), polychlorinated biphenyls, polycyclic aromatic hydrocarbons (PAHs), and other contaminants. These pollutants pose potential threats to the diverse array of organisms inhabiting mangroves, including aquatic animals, plants, and microorganisms [11,12]. In recent years, POPs in mangrove ecosystems have emerged as a significant environmental concern and have garnered increasing attention from researchers [11,13]. Among POPs, PBDEs, such as tetrabromodiphenyl ether (BDE-47) and decabromodiphenyl ether (BDE-209), are extensively utilized in electronic products, textiles, and household building materials. Consequently, they are easily dispersed into the environment and migrate to coastal areas, such as mangrove sediments, through various pathways [14].

To date, PBDEs have been widely found in mangrove sediments [15,16,17,18]. Studies conducted in the Hainan Province of China revealed that the concentrations of PBDEs (sum of ten PBDE congeners: BDE-28, 35, 47, 77, 99, 100, 153, 154, 183, and 209) in sediments ranged from 83 to 2929 pg g^−1^ dw, with an average concentration of 713 pg g^−1^ dw. Specifically, the Dongzhai Port National Nature Reserve recorded concentrations of 741 pg g^−1^ dw, while Sanya Bay and Yalong Bay recorded concentrations of 639 pg g^−1^ dw and 590 pg g^−1^ dw, respectively [11]. The noteworthy aspect is that PBDEs exhibit bioaccumulative, persistent, and multifaceted biotoxic effects [19]. Previous research has found that the concentration of PBDEs (sum of ten PBDE congeners) in mangrove plant tissues was 675 ± 4441 pg g^−1^ dw, with average concentrations of 1048, 498, 546, and 364 pg g^−1^ dw, respectively, in the leaves, branches, roots, and fruits [11]. These results indicate that PBDEs can accumulate in mangrove plant tissues. In addition, the microorganisms found in mangrove sediments are capable of degrading highly brominated homologues, such as BDE-209, into less brominated homologues, like BDE-47. These low-brominated congeners are more readily absorbed by organisms [20] and plants [21], which may amplify the ecological risk associated with PBDEs in the environment. BDE-47 has been shown to be one of the ubiquitous PBDE congeners in mangrove sediments, especially in the Southeast coastal areas of China [22].

Stomata are natural pores on the surface of plant leaves, which function as channels for water exchange during transpiration and gas exchange during photosynthesis [23]. In response to external stimuli, plants can modulate stomatal aperture and/or stomatal density to adjust their physiological activities and cope with adverse environmental conditions [23,24,25]. Research has shown that various environmental stimuli, such as drought [26], salinity [27], pollution including heavy metals [28], microplastics [29], industrial wastewater [30], and PAHs [31], can influence stomatal characteristics, thereby regulating plant leaf physiology and morphology. Furthermore, the degree and density of stomatal opening/closing also govern gas exchange within the leaves, ultimately impacting the plant photosynthetic efficiency [32]. The process of plant photosynthesis relies on the utilization of light energy through the presence of light-harvesting chlorophyll a/b-binding proteins (LHC) in both photosystem I (PSI) and photosystem II (PSII). Two major gene groups comprising the LHC protein complex are LHCI (encoded by the LHCA gene family) and LHCII (encoded by the LHCB gene family), which are closely associated with the PSI and PSII photosystem core protein complex, respectively [33]. Previous studies have demonstrated that diverse pollutants exert their toxic effects on photosynthetic organs through distinct mechanisms of photosynthetic toxicity [34,35,36]. In addition, the accumulation of BDE-47 in plant leaves can result in direct toxicity to the leaves, thus inhibiting the photosynthetic activity of plants [37,38]. It has been reported that the inhibitory effects of BDE-47 on plant photosynthetic activity is mediated through the regulation of the functionality of chloroplast PSI and PSII [39], including the inhibition of chlorophyll synthesis, the disruption of chloroplast structure [40], and interruption of chloroplast electron transfer and ROS production [22]. In contrast to terrestrial plants, mangrove plants grow in harsh tropical intertidal environments where photosynthesis can reach light saturation. Thus, the structures and function of stomata and chloroplasts in leaves are essential for them to adapt to such demanding surroundings. However, whether BDE-47 affects stomatal characteristics and the photosynthesis process in mangrove plant species is unknown. The use of chlorophyll fluorescence to monitor photosynthetic performance in plants is now widespread [41]. In addition, gas exchange parameters, including net photocondition rate (*P_n_*), transpiration rate (*T_r_*), stomatal conductance (*g_s_*), and intercellular carbon dioxide concentration (*C_i_*), can reveal the tolerance of plants to external conditions, such as salinity [42], temperature [43], and hydration [44]. Therefore, we hypothesized that BDE-47 might disturb stomatal characteristics (including stomatal density and status and gas exchange parameters) and photosynthesis (chlorophyll fluorescence, pigment contents, and chloroplast structure). Furthermore, despite the potential photosynthetic toxicity of BDE-47, the key pathways and genes that interact with BDE-47 are also not clearly understood.

The *K. obovate* is a woody plant predominantly distributed from East Asia to Southeast Asia [45]. It is the most common mangrove plant along the coastline in South China and can grow up to 3 m tall. Due to its wide distribution, important ecological functions, and the characteristics of the easy cultivation of its seedlings in the laboratory, *K. obovate* seedlings have become the subject of increasing research, with numerous studies exploring its various aspects [46,47,48,49]. Therefore, in this study, *K. obovate* seedlings were used to investigate the effects of BDE-47 at different environmental concentrations (0, 50, 500, and 5000 ng g^−1^ dw) on the stomatal characteristics and photosynthesis. Because RNA sequencing is an effective and extensively used tool to investigate the genetic basis of stress tolerance mechanisms in plants [50], it was employed to reveal the key pathways and genes involved in photosynthetic toxicity induced by BDE-47 exposure. The toxicity data obtained in this study could provide preliminary information for understanding the risk of BDE-47 on mangrove plants.

## 2. Materials and Methods

### 2.1. Chemicals

The chemical BDE-47 used was purchased from Macklin Company (Shanghai, China) with a purity of 97%. Analytical-grade toluene was obtained from the Guangzhou Chemical Reagent Company (Guangzhou, China).

### 2.2. Soil Preparation

The experimental soil consisted of river sand, red soil, and nutrient soil in a mass ratio of 2:2:1. After natural air drying, it was filtered through a 2 mm mesh. The preparation of BDE-47-exposed soil followed previous research methods [51,52]. Initially, BDE-47 was dissolved in toluene, and stock solutions with concentrations of 0, 0.01, 0.1, and 1 mg mL^−1^ were prepared. Then, 40 mL of the stock solution was added to each batch of air-dried soil (100 g) and thoroughly mixed before being dried in a dark environment for 48 h to remove the toluene solvent. Subsequently, the treated soil (100 g) was mixed with an additional amount (7.9 kg) of air-dried soil to obtain different concentrations of BDE-47-exposed soil. The exposed soil with different concentrations of BDE-47 was divided into separate square basins (40 cm × 30 cm × 20 cm, with a drainpipe at the bottom). These samples were then aged at room temperature away from the light for a duration of 30 d while being stirred every 7 d to ensure the even distribution of BDE-47 within the soil.

### 2.3. Plant Exposure and Sampling

The experiment was conducted in July 2023 in a plant greenhouse (temperature: 28–35 °C, humidity: 55–75%, and light: 600–1200 μmol m^−2^ s^−1^) located at Hainan Normal University in Haikou, China. *K. obovata* seedlings were obtained from a clean and pollutant-free artificial mangrove base in Maoming City (Guangdong Province, China) and pre-cultured in a nutrient solution with a salinity of 10‰ for 7 days. Healthy 5-month-old seedlings at the 3-leaf stage with similar sizes were randomly selected. Then, the fresh weights, plant heights, and stem diameters were measured with a balance, a ruler, and vernier calipers, respectively. The morphological measurements were 11.83 ± 0.66 g for fresh weights, 24.20 ± 0.91 cm for plant heights, and 9.97 ± 0.17 mm for stem diameters. They were then transplanted into basins containing eight-kilogram exposed soil with different concentrations of BDE-47, with six plants planted in each basin. The different exposure groups with different concentrations of BDE-47 were named as L0 (0 ng g^−1^ dw), LT50 (50 ng g^−1^ dw), LT500 (500 ng g^−1^ dw), and LT5000 (5000 ng g^−1^ dw). Each group comprised 4 biological replicates with the same exposed soil. The plants were continuously cultivated with natural light and ventilation while adding salt water with a salinity of 10‰ every 5 d to maintain water stability. At the 30th day, the position of the basins was changed over to avoid the effects of light direction [53]. After 60 d of exposure, the gas exchange parameters and fluorescence parameters of the living leaves were first measured (methods are described below). Subsequently, the plants were carefully removed from the soil and their roots were cleaned using salt water with a salinity of 10‰. Photographs were taken with a Nikon camera (Nikon D5600, Japan Optical Industry Co., Ltd., Tokyo, Japan) on a black background cloth (0.1 cm precision steel rule for reference). The fresh weight, plant height, and diameter of stem were measured. The leaf samples were collected for electron microscopy or snap-frozen in liquid nitrogen and stored at −80 °C for biochemical analysis and RNA extraction.

### 2.4. Measurement of Stomatal Characteristics and Gas-Exchange Parameters 

The leaves were randomly selected and washed with phosphate-buffered saline (PBS). The detection of stomatal characteristics on the lower epidermis followed the methods used in a previous study with slight modifications [54]. Briefly, tissue pieces smaller than 5 mm × 5 mm were carefully excised from the central region of the leaf using a scalpel to avoid damaging the veins. A triangular incision was made in the upper right corner of each tissue piece to indicate the side (as stomata are exclusively present on the lower epidermis of leaves). The samples were promptly immersed in a solution containing 2.5% glutaraldehyde and then fixed at 4 °C. After fixation, the samples were rinsed with PBS, dehydrated using an ethanol gradient, replaced with pure tert-butanol, and freeze-dried for a duration of 12–24 h. Finally, a metal conductive layer (JEOL, JFC-1600 Auto Fine Coater, Tokyo, Japan) was sprayed onto the dried tissues, and the stomata were observed under a beam voltage of 5 kV using a field emission scanning electron microscope (SEM, JSM-7100F, JEOL, Tokyo, Japan). The SEM magnification was set at 200× with a field of view measuring approximately 0.2826 mm^2^ (1280 × 1024 pixels), and then converted into the number of stomata in an area of 1 mm^2^ as the stomatal density (number of pores per unit area). For each exposure group, five leaves were randomly chosen and three fields of view were captured for analysis using Image J software 1.54j (National Institutes of Health, Stapleton, NY, USA) to determine the stomatal density (number of stomata per unit area) and stomatal state (number of stomata at different opening sizes). The degree of stomatal opening was defined as: fully open (FO), semi-close (SC), and fully closed (FC). The stomatal opening ratio was calculated as the proportion of stomata with different opening degrees (FO, SC, and FC) to the total stomata, and the fields were chosen according to the described method used for the stomatal density.

The gas exchange parameters, including *P_n_* (μmol m^−2^ s^−1^), *T_r_* (mmol m^−2^ s^−1^), *g_s_* (mmol m^−2^ s^−1^), and *C_i_* (μmol mol^−1^), were measured using the CI-340 handheld Photosynthesis system (CID Inc., Camas, WA, USA) on a sunny morning between 9:00 and 11:00 [55]. For each exposure concentration, plants were randomly selected, and the second pair of mature leaves at the top of each plant was chosen for measurement (*n* = 12). The measurement conditions were: CO_2_ concentration of 450 μmol mol^−1^, light intensity of 1000 μmol m^–2^ s^–1^, temperature of 35 °C, and air relative humidity of 65%. When the instrument readings were stable, the parameters were recorded.

### 2.5. Chloroplast Structure Observation

The chloroplast ultrastructure of the leaves was observed by transmission electron microscopy, following a previously reported method [56]. Firstly, small tissue fragments were excised from comparable leaf regions, fixed with 2.5% (*v*/*v*) glutaraldehyde, and trimmed to a size of 1 mm^3^. Then, the samples were subjected to fixation using 1% (*w*/*v*) osmic acid, rinsing, gradient dehydration with ethanol, gradual penetration with acetone, and embedding in epoxy resin (SPI-PonTM 812). Ultra-thin sections with a thickness of 70 nm were prepared using the Leica EM UC7 ultramicrotome (Leica Microsystems IR GmbH, Wetzlar, Germany) and mounted on a 150-mesh cuprum grid with a formvar film. Staining was performed using solutions containing 2% uranyl acetate and 2.6% lead citrate. Finally, the scanning of the sections was conducted utilizing a Hitachi transmission electron microscope (HT7800, Hitachi Ltd., Tokyo, Japan). Three to five cells and their chloroplast organelles were imaged in each exposure concentration.

### 2.6. Pigment and Fluorescence Parameter Assays 

The determination of chlorophyll (Chl a and Chl b) and carotenoid (Car) contents was conducted following a previously reported method with slight modifications [57,58]. Approximately 0.2 g of fresh leaves were finely chopped and subsequently immersed in 95% ethanol under dark conditions for approximately 48 h until decolorized. After centrifugation, the absorbance of the supernatant was measured at the wavelengths of 470, 649, and 665 nm using a multifunctional microplate reader (SpectraMax iD, SAN Jose, CA, USA). Subsequently, the parameter Chl a+b was calculated (*n* = 3).

The fluorescence parameters were measured using a pulse-modulated chlorophyll fluorometer (MINI-PAM-II, Walz, Effeltrich, Germany). After acclimating the leaves in darkness for 30 min, the minimal fluorescence (Fo) and maximal fluorescence (Fm) were recorded to calculate the maximum quantum yield of PSII according to the formula [PSII(Fv/Fm) = (Fm − Fo)/Fm] [59]. The maximum fluorescence yield (Fm’) under light-adapted conditions and steady-state fluorescence yield (F’) under light steady-state conditions were recorded as well. The quantum yield efficiency Y(II) of PSII in light-adapted leaves was calculated as Y(II) = (Fm’ − F’)/Fm [60]. The determination time and leaf examining location were consistent with the method that was used for the gas exchange parameter measurement (*n* = 12).

### 2.7. Transcriptome Sequencing Analysis

Four leaves were used for RNA extraction, reverse transcription, and transcriptome sequencing. Transcriptome sequencing was performed using the platform of Novogene Bioinformatics Technology Co., Ltd. (Beijing, China). The raw data were filtered to obtain clean data with high quality. These data were aligned with the published reference genomes of *K. obovata* (https://download.cncb.ac.cn/gwh/Plants/Kandelia_obovata_Kandelia_candel_GWHACBH00000000.1/GWHACBH00000000.1.genome.fasta.gz, accessed on 21 March 2024) for sequence alignment and functional annotation [13]. The software Hisat2 (v2.0.5, Bioconductor, Wako, Japan) and featureCounts (v1.5.0-p3, Bioconductor, Wako, Japan) were used for sequence alignment and gene quantification, respectively. Gene expression levels were determined in terms of fragments per kilobase of transcript sequence per million base pairs-sequenced (FPKM) values using the RSEM (v1.2.15, Bioconductor, Wako, Japan) software, and differentially expressed genes (DEGs) were detected using the DESeq2 (v1.20.0, Bioconductor, Wako, Japan) software, with a *padj* < 0.05 and |log_2_FC| > 1 as the thresholds for significance.

The obtained DEGs were further visualized and mapped. A gene ontology (GO) (http://www.geneontology.org/, accessed on 21 March 2024) functional enrichment analysis and the Kyoto Encyclopedia of Genes and Genomes (KEGG) (https://www.kegg.jp/kegg/, accessed on 21 March 2024) pathways enrichment analysis were performed using clusterProfiler software (v3.8.1, https://www.bioconductor.org/packages/release/BiocViews.html#___Software, accessed on 21 March 2024). A principal component analysis (PCA) and co-expression Venn diagram analysis were performed using ggplot2 package(Hadley Wickham, Hamilton, New Zealand) and VennDiagram package of R (v3.0.3, Hadley Wickham, Hamilton, New Zealand), respectively. All cluster heat map drawing was based on the ggplot2 package of the Novogene cloud platform and TBtools-II (v2.056). Hub genes were screened by PPI co-expression network map analysis by the cytoscape software (v3.9.1). The significance of KEGG terms and pathways was corrected using *padj* (*padj* < 0.05) with a strict threshold, and the visualization map with enrichment function was generated after the normalization of DEGs.

### 2.8. qRT-PCR Analysis

The primers were designed based on the primer 5.0 (Premier Biosoft, Palo Alto, CA, USA) software, and primer synthesis was performed by Sangon Bioengineering (Shanghai, China) Co., Ltd. After RNA extraction and cDNA synthesis, qRT-PCR was performed using an Aria MX real-time PCR system (Agilent Technologies, Inc., Santa Clara, CA, USA). The qRT-PCR primers used are shown in the Supplementary Table S1. The relative gene expression levels were determined by the 2^−∆∆CT^ method using 18SrRNA from *K. obovata* as an internal reference gene [61].

### 2.9. Statisical Analysis

Statistical analyses were conducted using Excel v.2019 (Microsoft Excel, Redmond, WA, USA). All data were analyzed for normality (Kolmogorov–Smirnov test) and homogeneity of variance (Levene’s test). Statistical significance was assessed by a one-way ANOVA followed by least significant difference (LSD) or a non-parametric Kruskal–Wallis test at *p* < 0.05 for all the tests in SPSS v.28.0 (IBM Corp., Armonk, NY, USA). The graphs were created using GraphPad Prism (v8.00, GraphPad Software, San Diego, CA, USA), and all data are displayed as the mean ± standard error (S.E.). Significant differences are indicated by letters on the figures.

## 3. Results

### 3.1. Effects of BDE-47 Exposure on the Growth of K. obovata

**Figure 1:**
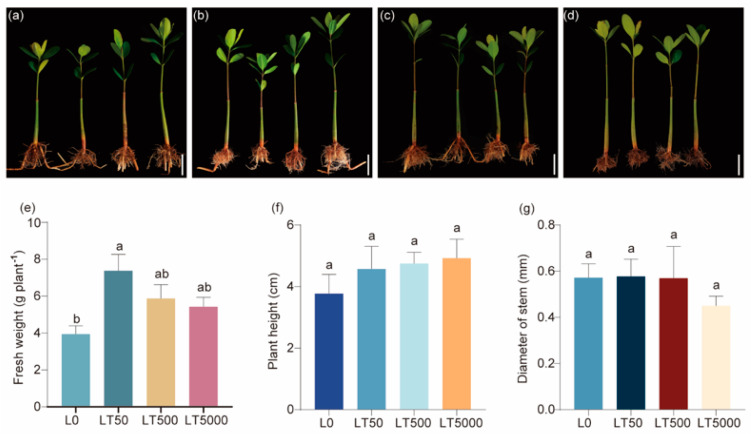
Effects of BDE-47 (0, 50, 500, and 5000 ng g^−1^ dw) soil exposure for 60 d on the growth of *K. obovate*. (**a**–**d**) Plant morphology in L0 (0 ng g^−1^ dw, **a**), LT50 (50 ng g^−1^ dw, **b**), LT500 (500 ng g^−1^ dw, **c**), and LT5000 (5000 ng g^−1^ dw, **d**) (bar = 5 cm). (**e**) Fresh weight. (**f**) Height. (**g**) Stem diameter. Data are expressed as mean ± S.E., with different lowercase letters indicating significant differences between the groups (*p* < 0.05, *n* = 4).

No significant changes were observed in morphology following exposure to BDE-47 (Figure 1a–d). In comparison to L0, the fresh weights in the LT50 exhibited a significant increase (1.868-fold; *p* = 0.004), while no significant difference was found in the LT500 and LT5000 (Figure 1e). In addition, there was no significant effect of the different concentrations of BDE-47 on the height and stem diameters (Figure 1f,g).

### 3.2. Effects of BDE-47 Exposure on the Stomata on Leaves of K. obovata

**Figure 2:**
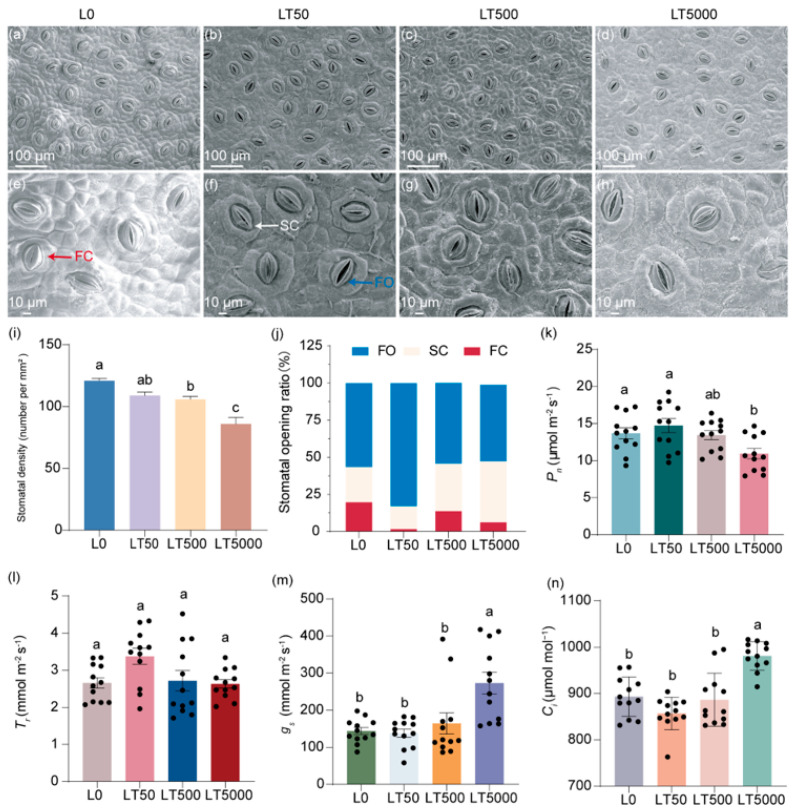
Effects of BDE-47 (0, 50, 500, and 5000 ng g^−1^ dw) soil exposure for 60 d on the stomatal characteristics and gas exchange parameters of *K. obovata*. (**a**–**h**) The leaf stomatal states in L0 (0 ng g^−1^ dw), LT50 (50 ng g^−1^ dw), LT50 (500 ng g^−1^ dw), and LT5000 (5000 ng g^−1^ dw); FO: fully open state (blue arrow), SC: semi-close state (white arrow), FC: fully closed state (red arrow). (**i**) Stomatal density (*n* = 15). (**j**) Stomatal opening ratio. (**k**) *P_n_* (*n* = 12). (**l**) *T_r_* (*n* = 12). (**m**) *g_s_* (*n* = 12). (**n**) *C_i_* (*n* = 12). Data are expressed as mean ± S.E., with different lowercase letters indicating significant differences between the groups (*p* < 0.05) and the points on the bar graph indicate the degree of dispersion of duplicate samples.

The stomatal characteristics of the lower epidermis of *K. obovata* leaves are illustrated in (Figure 2a–h), demonstrating a decrease in the stomatal density and an increase in the extent of stomatal aperture after exposure to BDE-47. By quantifying the stomatal density per mm^2^, it was shown that, compared to L0, LT50, LT500, and LT5000 exhibited a dose-dependent reduction in the stomatal density by 8.32%, 15.31%, and 20.60%, respectively (Figure 2i); significant differences were found for LT500 and LT500 (*p* = 0.008 and *p* < 0.001, respectively) (Figure 2i). Based on their degree of opening, the stomata were categorized as in fully open (FO), semi-close (SC), or fully closed states (FC), as indicated in Figure 2e,f. In L0, the proportions of FO, SC, and FC states were 57.7%, 24.6%, and 17.8%, respectively. However, these proportions shifted to 81.9%, 15.7%, and 2.5% in LT50 and 31.6%, 55.7%, and 12.6% in LT500 for the FO, SC, and FC states, respectively. They changed to 51.4% and 41.4% for the FO and FC states, respectively, and no presence of the SC state was observed in LT5000 (Figure 2j). Compared to L0, the proportion of total FO and SC states increased by 15.3%, 5.1%, and 10.6% in LT50, LT500, and LT500, respectively (Figure 2j).

No significant difference in *P_n_* was found in LT50 and LT500 compared to L0; however, *P_n_* exhibited a significant decrease of 20.03% in LT5000 (*p* = 0.014; Figure 2k). For *T_r_*, no significant difference was found in all exposed groups when compared to L0 (Figure 2l). For *g_s_* and *C_i_*, no significant difference was found in LT50 and LT500 compared to L0; however, *g_s_* and *C_i_* were increased significantly by 89.63% (*p* < 0.001) and 9.82% (*p* < 0.001), respectively, in LT5000 compared to L0 (Figure 2m,n).

### 3.3. Effects of BDE-47 Exposure on the Chloroplast Ultrastructure in Leaves of K. obovata

**Figure 3:**
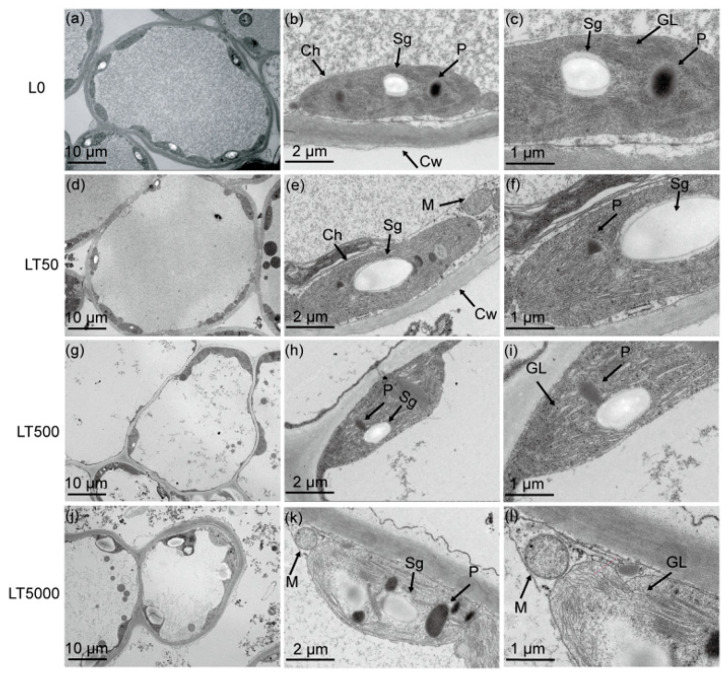
Effects of BDE-47 (0, 50, 500, and 5000 ng g^−1^ dw) soil exposure for 60 d on the chloroplast ultrastructure of *K. obovata*. (**a**–**c**) L0 (0 ng g^−1^ dw), (**d**–**f**) LT50 (50 ng g^−1^ dw), (**g**–**i**) LT50 (500 ng g^−1^ dw), and (**j**–**l**) LT5000 (5000 ng g^−1^ dw). Ch: chloroplasts, M: mitochondria, Cw: cell wall, Sg: starch grain, P: plastoglobulus, GL: grana lamella.

In L0 (Figure 3a–c), the leaf cells exhibited a complete and nearly spherical morphology, with an abundance of elliptical intracellular chloroplasts. The internal chloroplast lamella appeared smooth, while the size and quantity of starch grains and plastoglobulus were moderate. No significant differences in cell and organelle structure were observed in LT50 compared to L0 (Figure 3d,e). However, in LT500, the leaf cells started to exhibit folding and deformation (Figure 3i). Furthermore, notable changes occurred, including the disintegration of chloroplasts, the expansion of starch grains, as well as an increase in plastoglobulus count, in LT5000 (Figure 3j–l). Additionally, the thylakoid grana lamellar structure of the chloroplasts became relaxed in L500 and further vacuolated in L5000 (Figure 3i,l).

### 3.4. Effects of BDE-47 Exposure on the Pigments and Fluorescence Parameters in Leaves of K. obovata

**Figure 4:**
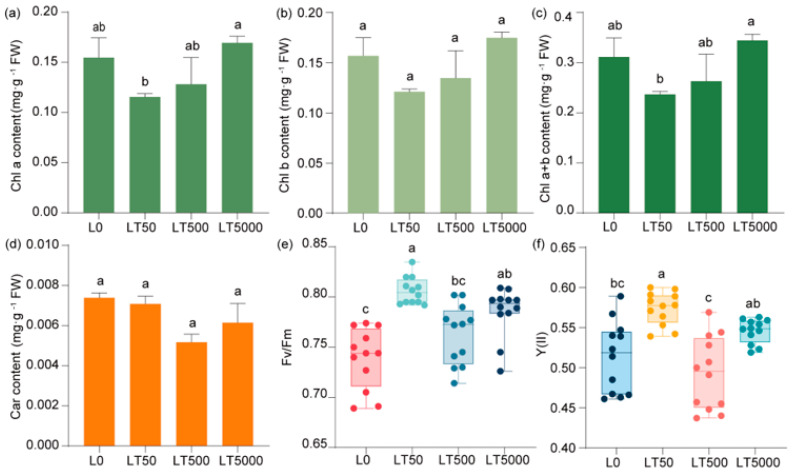
Effects of BDE-47 (0, 50, 500, and 5000 ng g^−1^ dw) soil exposure for 60 d on the pigments and fluorescence parameters of *K. obovata*. (**a**) Chl a, (**b**) Chl b, (**c**) Chl a+b, (**d**) carotenoid, (**e**) Fv/Fm, and (**f**) Y(II). Data are expressed as mean ± S.E., with different lowercase letters indicating significant differences between exposed groups (*n* = 12, *p* < 0.05) and the points on the bar graph indicate the degree of dispersion of duplicate samples.

Compared to L0, no significant difference was observed in the contents of chlorophyll (Chl a, Chl b, and Chl a+b) (Figure 4a–c) and carotenoids (Figure 4d) in the BDE-47-exposed group. In comparison to L0, Fv/Fm and Y(II) exhibited an initial increase followed by a decrease and then another increase with the increase in exposure concentrations. In LT50, both Fv/Fm and Y(II) showed significant increases (1.091-fold and 1.118-fold; *p* < 0.001 and *p* < 0.001, respectively). Compared to L0, Fv/Fm and Y(II) did not show a significant increase in LT500, but there was a significant increase in Fv/Fm (1.041-fold; *p* = 0.027) in LT5000, while no significant change was found for Y(II) (Figure 4e,f).

### 3.5. Transcriptomic Analysis and qPCR Verification

**Figure 5:**
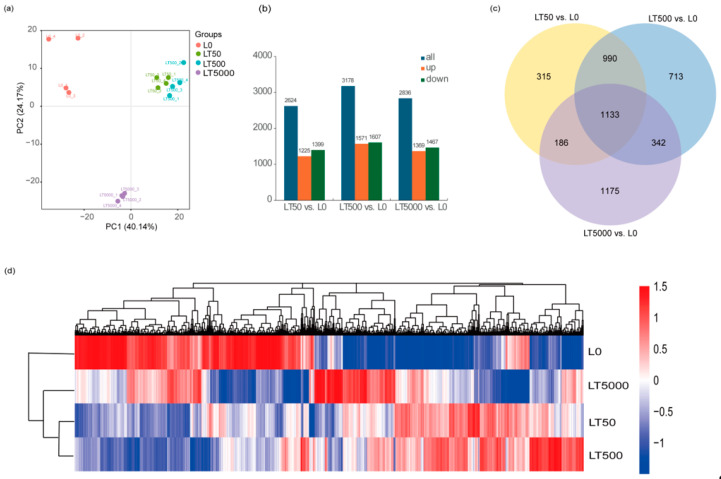
Transcriptome analysis of the leaves of *K. obovata* after 60 d of soil exposure to BDE-47 (0, 50, 500, and 5000 ng g^−1^ dw). (**a**) PCA; (**b**) quantitative analysis of DEGs; (**c**) Venn analysis of DEGs; and (**d**) clustering heat map of DEGs.

A PCA (principal component analysis) was conducted on the FPKM of different BDE-47 exposure groups. The results reveal that, in PC1 (48.25%), the exposure groups were dispersed among the different concentrations, indicating significant differences among exposed groups. However, the LT50 and LT500 groups exhibited a closer proximity, suggesting minimal differences. In the PC2 (26.28%) outcomes, L0 displayed a positive dispersion away from both LT50 and LT500, while it showed a negative dispersion away from LT5000, signifying substantial distinctions between LT5000 and other exposed groups. Most samples within each exposure group formed tight clusters, indicating minimal variations among them (Figure 5a). A total of 110.61 GB of high-quality clean reads (Q30 > 95%) were obtained through sequencing. Compared to L0, the differential expression gene analysis identified a total of 4854 DEGs, with 1225 DEGs up-regulated and 1399 DEGs down-regulated in LT50; there were 1571 up-regulated DEGs and 1607 down-regulated DEGs in LT500, and 1369 up-regulated DEGs and 1467 down-regulated DEGs in LT5000 (Figure 5b). The Venn diagram results reveal that three different concentrations of BDE-47 co-affect a total of 1133 DEGs, accounting for approximately 23.34% of all 4854 identified DEGs. Furthermore, with the increase in BDE-47 exposure concentrations, the number of specific DEGs increased (Figure 5c).

In addition, the clustering analysis of the DEGs showed similar results to the PCA. DEG expression patterns were different between L0 and each BDE-47 exposure group, whereas the clustered heat maps of LT50 and LT500 had similar DEG expression patterns (Figure 5d).

**Figure 6:**
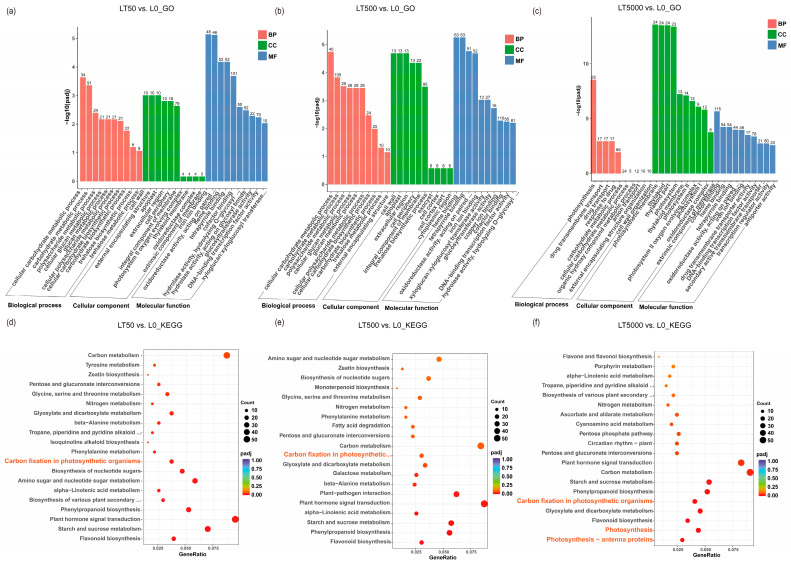
GO and KEGG analysis of the transcriptome in *K. obovata* leaves after 60 d of soil exposure to BDE-47 (0, 50, 500, and 5000 ng g^−1^ dw). (**a**–**c**) GO enrichment analysis of LT50 vs. L0, LT500 vs. L0, and LT5000 vs. L0. (**d**–**f**) KEGG enrichment analysis of LT50 vs. L0, LT500 vs. L0, and LT5000 vs. L0.

The identified DEGs were subjected to GO and KEGG enrichment analysis. In comparison to L0, LT50 exhibited an enrichment of 995 DEGs in GO, with 352 up-regulated and 643 down-regulated DEGs. Similarly, LT500 showed an enrichment of 1227 DEGs, with 485 up-regulated and 742 down-regulated DEGs. Furthermore, LT5000 displayed an enrichment of 1510 DEGs, with 619 up-regulated and 891 down-regulated DEGs. Among the three exposure groups of LT50, LT500, and LT5000 (Figure 6a–c), the transmembrane transporter activity (GO:0022857), cellular carbohydrate metabolic process (GO:0044262), polysaccharide metabolic process (GO:0005976), and photosynthesis (GO:0015979) were significantly enriched in the biology process (BP) category, while cell wall (GO:0005618), external encapsulating structure (GO:0030312), apoplast (GO:0048046), and extracellular region (GO:0005576) were significantly enriched in the cellular component (CC) category. Additionally, the photosynthetic membrane (GO:0034357), thylakoid membrane (GO:0042651), photosystem I (GO:0009522), photosystem II (GO:0009523), and oxidoreductase complex (GO:199 0204) were found to be significantly enriched in the CC category in LT5000 vs. L0. Among the three exposure groups of LT50, LT500, and LT5000, iron ion binding (GO:0005506), heme binding (GO:0020037), tetrapyrrole binding (GO:0046906), transcription regulation activity (GO:0140110), xyloglucosyl transferase activity (GO:0016762), and oxidoreductase activity (GO:0016705) were identified in the molecular function (MF) category. Furthermore, the KEGG pathway analysis revealed a total of 47 significantly enriched pathways involving 1056 DEGs. The top pathways (Figure 6d–f) included flavonoid biosynthesis (mp000941); starch and sucrose metabolism (mp00500); plant hormone signal transduction (mp04075); phenylpropanoid biosynthesis (mp00940); alpha-linolenic acid metabolism (mp00592); cutin, suberine, and wax biosynthesis (mp00073); amino sugar and nucleotide sugar metabolism (mp00520); photosynthesis antenna proteins (mp00196); photosynthesis (mp00195); carbon fixation in photosynthetic organisms (mp00710); and carbon metabolism (mp01200), etc. Overall, LT5000 exhibited more enriched items or metabolic pathways in GO or KEGG compared to LT50 and LT500.

**Figure 7:**
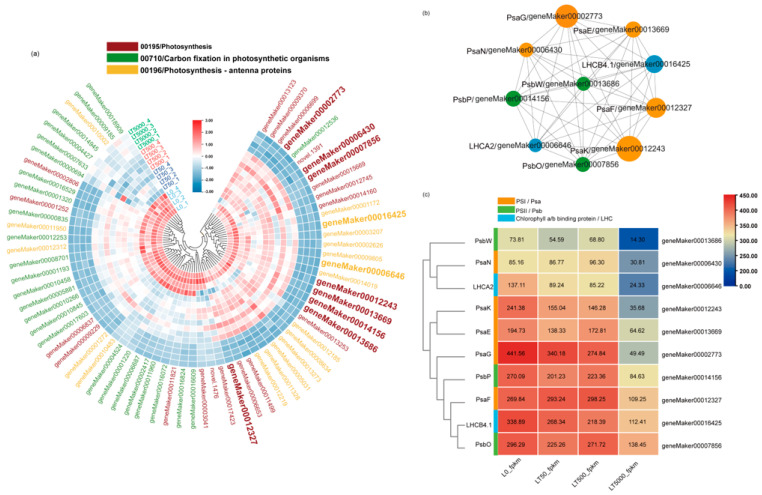
Heat map and co–expression network analysis of genes associated with photosynthetic pathways in *K. obovata* leaves after 60 d of soil exposure to BDE-47 (0, 50, 500, and 5000 ng g^−1^ dw). (**a**) Heat maps depicting the co–expression patterns of genes involved in photosynthetic pathways. (**b**) Protein–protein interaction (PPI) screening for hub genes (Top 10). (**c**) FPKM changes in Top10 hub genes.

Furthermore, in all groups, a total 70 down-regulated DEGs associated with plant photosynthesis were enriched in three down-regulated pathways. Among the 70 DEGs, 9 DEGs were associated with PSI (Psa), 11 DEGs were associated with PSII (Psb), 19 DEGs were related to chlorophyll a/b-binding protein (LHC), and 3 DEGs were related to ATP synthase. In both LT50 and LT500, 19 identical DEGs in carbon fixation in photosynthetic organisms (mp00710) showed significant down-regulation, while in LT5000, 18 DEGs in photosynthesis antenna proteins (mp00196), 26 DEGs in photosynthesis (mp00195), and 25 DEGs in carbon fixation in photosynthetic organisms (mp00710) were significantly down-regulated (Figure 7a) (Table S2 in Supplementary Materials). 

A PPI co-expression network analysis was conducted to identify hub genes, from which the Top10 genes were obtained, including geneMaker00012327 (*PsaF*), geneMaker00014156 (*PsbP*), geneMaker00012243 (*PsaK*), geneMaker00006430 (*PsaN*), geneMaker00016425 (*LHCB4.1*), geneMaker00013669 (*PsaE*), geneMaker00002773 (*PsaG*), geneMaker00006646 (*LHCA2*), geneMaker00007856 (*PsbO*), and geneMaker00013686 (*PsbW*) (Figure 7b). The FPKM values of these 10 DEGs are shown in Figure 7c. The analysis showed that eight of these DEGs were present in the photosynthesis pathway (mp00195), which is related to the functions of PSI and PSII. The other two DEGs belong to the photosynthesis antenna proteins pathway (mp00196), which belongs to the LHC gene family and is related to chlorophyll a/b-binding proteins. Finally, the Top10 genes were analyzed by qPCR, and the results are consistent with the RNA-seq analysis (Figure S1 in Supplementary Materials).

## 4. Discussion

This study found that BDE-47 reduced the stomatal density and photosynthetic capacity in the leaves of the mangrove species *K. obovate*. Furthermore, the transcriptome sequencing and bioinformatics analysis revealed that the photosynthesis (mp00195) and photosynthesis antenna proteins (mp00196) pathways as well as their related genes might be the key targets of BDE-47 in photosynthesis in *K. obovata*.

Stomata play a crucial role in plant growth and stress resistance [23,62]. The stomata of *K. obovata* specifically exist in the lower epidermis of the leaves [63]. This study demonstrated that BDE-47 suppressed the stomatal density in the lower epidermis while promoting the number of semi-open stomata, indicating a stress response mechanism employed by the plants under adverse conditions. Generally, there is a negative correlation between stomatal density and the extent of opening or closing, allowing plants to adapt to unfavorable environments through effective stomatal control [26,32]. In this study, the exposure to a high concentration of BDE-47 resulted in a significant decrease in *P_n_* and a significant increase in *C_i_*, thereby inducing leaf light suppression. Similar phenomena were also observed in salt-stressed plants, where increased salt stress led to a significant reduction in plant *P_n_* and *T_r_* as well as an increase in *C_i_* [23,64]. It has been reported that leaves with a higher stomatal aperture exhibit elevated *g_s_* and *T_r_* [53]. However, no significant change was observed in *T_r_* after BDE-47 exposure in this study, which may be attributed to the extensive leaf sclerification and enhanced water retention capacity of the plants. This water–air tradeoff strategy employed by the plants ensures the efficient utilization of carbon substrates and water for photosynthetic product synthesis while minimizing the risk of hydraulic failure [62,65]. Furthermore, it was found that *P_n_* significantly decreased while *g_s_* significantly increased in this study, which differs from the effects of other pollutants on plants. For instance, a previous study found that tetrabromobisphenol A (TBBPA) directly caused substantial declines in *P_n_*, *T_r_*, *g_s_*, and other photosynthetic parameters by reducing CO_2_ uptake in tobacco leaves [66]. We speculate that mangrove plants may enhance their photosynthetic performance through the modulation of stomatal opening–closing dynamics to facilitate a greater CO_2_ absorption; however, further investigation is required.

The results obtained from the transmission electron microscopy revealed that exposure to BDE-47 had detrimental effects on the structure of chloroplasts, leading to the evident damage and disruption of thylakoid lamella structure, leading to a marked damage and destruction of the thylakoid lamina structure, which significantly affected leaf development and resulted in morphological changes. Various stresses, such as pollutants and drought, can induce different signals within plants, triggering the physiological regulation of the plant system and causing physical harm [67]. Our findings suggest that soil exposure to BDE-47 may modify leaf physiology, biochemistry, and molecular status by altering root signaling and transmission to leaves. In addition, increases in starch grains and plastoglobulus, potent antioxidants present in plant cells [68], were observed in this study. These substances can enhance metabolic activities within chloroplasts while preventing further lipid peroxidation production in cells. As a result, they can reduce or alleviate damage to cell structures [69,70].

Chlorophyll serves as the foundation for plant photosynthesis [71]. In this study, varying concentrations of BDE-47 exposure did not yield significant impacts on the pigment contents of leaves. This finding deviates from previous results, potentially attributed to differences in exposure methods and the lower concentrations of BDE-47 used. Previous studies have found that lower concentrations of BDE-47 (0.1 and 1 mg L^−1^) had no effect on chlorophyll contents in hydroponic *K. obovata* seedlings, whereas high concentrations (5 and 10 mg L^−1^) significantly hindered seedling growth, resulting in leaf loss and a substantial reduction in Chl a levels [22]. Furthermore, BDE-47 inhibits Chl a synthesis in tobacco (*Nicotiana tabacum* L.) [39]. Although chlorophyll levels were not significantly affected by BDE-47 exposure in this study, the transcriptome sequencing analysis revealed the inhibition of nearly all genes associated with the photosynthesis pathway, indicating a molecular-level interference within photosynthesis caused by BDE-47. We will continue this discussion in a later section.

The process of photosynthesis exhibits a high degree of responsiveness to environmental stress, with particular sensitivity observed in PSII, which is commonly employed as an indicator for assessing the response to environmental stressors [72,73]. Previous studies have demonstrated that exposure to BDE-47 can impair the photosynthetic capacity in plants, primarily characterized by a decline in photochemical activity and carbon sequestration ability [52,74]. In addition, the continuous exposure to BDE-47 impacted the efficiency and electron transport process of photosynthesis in *Lemna minor*, resulting in the inhibition of the maximum quantum yield (Fv/Fm) and performance index of PSII after a duration of 14 d [75]. Our experimental results show that, with the increase in the concentrations of BDE-47, Fv/Fm and PSII activities show a trend of decrease after the first increase. These results suggest that BDE-47 may promote light energy utilization and even enhance leaf photosynthetic function within a certain concentration range. However, previous studies found that, even at low concentrations (5–20 μg L^−1^), BDE-47 was toxic to algal growth, resulting in reduced Fv/Fm values, chlorophyll contents, and biomass levels [34]. In our results, the stimulation of photosynthesis at a low concentration of BDE-47 (50 ng g^−1^ dw) might be due to the toxic excitatory effects at a low concentration. However, with the increase in the concentration, the phototoxicity would be primary, ultimately resulting in the damage of the photosystem and the decrease in the photosynthetic capacity.

In this study, the potential molecular mechanism of BDE-47 exposure in photosynthesis in *K. obovata* leaves was further investigated using transcriptome sequencing. The KEGG pathway analysis revealed that gene expression in photosynthesis-related pathways was predominantly suppressed, and the inhibition was more obvious at the highest concentration (LT5000). Most of these suppressed genes were associated with the PSI and PSII photoreaction systems or the LHC photo-capture pigment membrane protein complex. Chloroplasts are the important sites of photosynthesis in plants [76]; therefore, a down-regulation of these photosynthetic genes implies a decline in plant photo-synthesizers. Similarly, previous studies have also reported that BDE-47 disrupts the PSI system in *Thalassiosira pseudonana* (a Marine diatom) [77] and inhibits the PSII system in duckweed [78], resulting in impaired light energy absorption. In addition, the GO enrichment analysis showed that the genes related to photosystem and chloroplast organelle membrane components were significantly down-regulated after 5000 ng g^−1^ dw BDE-47 exposure. We assumed that the chloroplast structure damage in *K. obovata* caused by BDE-47 exposure might be associated with the down-regulation of these photosynthetic membrane protein genes. In addition, the PPI enrichment results show that more PSI-related genes (*PsaF*, *PsaK*, *PsaN*, *PsaE*, and *PsaG*) were affected than those of PSII (*PsbP* and *PsbW*) and LHC (*LHCA2* and *LHCB4.1*), which indicates that the PSI photosystem might be more sensitive to BDE-47 exposure. Previous studies have also shown that tobacco is more sensitive to PSI activity but not to PSII activity when exposed to high concentrations of BDE-47 (50 μg/g dw) [39]. The different responses of PSI and PSII to pollutant stimuli may be due to the extremely slow repair of PSI during photo-damage, while PSII has a higher repair capacity, leading to a functional imbalance between the two photosystems [79]. However, photosynthesis in higher plants is a complex process. For the mangrove plant *K. obovate*, BDE-47 exposure at higher concentrations down-regulated the genes related to PSI and PSII and the light-harvesting chlorophyll protein, further indicating inhibition of photosynthesis by BDE-47 in *K. obovata*.

## 5. Conclusions

The results indicate that BDE-47 exposure can affect stomatal characteristics and the photosynthesis process in the mangrove plant species *K. obovata* seedlings, shown as the decrease in the stomatal density and the increase in opening degrees, damage in chloroplast ultrastructure, as well as the changes in gas exchange parameters. BDE-47 exposure did not yield significant impacts on the pigment contents, including chlorophyll, in the leaves. However, the photosynthesis at the molecular level was disturbed. We found that the photosynthesis (mp00195) and photosynthesis antenna proteins (mp00196) pathways might be the key pathways that BDE-47 inhibited related to the photosynthetic capacity of *K. obovata* seedlings. Additionally, PSI-related genes (*PsaF*, *PsaK*, *PsaN*, *PsaE*, and *PsaG*), PSII-related genes (*PsbP* and *PsbW*), and LHC genes (*LHCA2* and *LHCB4.1*) are the hub genes identified by PPI enrichment, which provide important toxic data for understanding the risk of BDE-47 to mangrove plants. Our results underscore the potential for organic pollutants (BDE-47) to compromise the photosynthetic performance of mangrove plants, thereby diminishing their ecological role as carbon sinks. In the future, mangrove pollution should be of great concern, and the implementation of measures aimed at mitigating the discharge of pollutants and remediation actions is imperative.

## Data Availability

Data will be made available upon request.

## Supplementary Materials

The following supporting information can be downloaded at: https://www.mdpi.com/article/10.3390/toxics12070456/s1. Table S1: Primers for real-time PCR; Table S2: KEGG database photosynthetic pathway gene annotation information; Figure S1: qRT-PCR results of the hub gene.
