# Supplementary material for: 2,2′,4,4′-Tetrabromodiphenyl Ether (BDE-47) at Environmental Levels Influenced Photosynthesis in the Mangrove Species Kandelia obovata
Source: Toxics. 2024 Jun 25;12(7):456. doi: 10.3390/toxics12070456 (PMC11281169; doi:10.3390/toxics12070456)
Supplement: Supplementary file 1 [file toxics-12-00456-s001.zip › Table S1.pdf]

Supplementary Table S1. Primer used for qRT-PCR analysis of *Kandelia obovate.*

gene_id	Primer pairs	
	Forward primer (5'-3')	Reverse primer (5'-3')
18S rRNA	GGGGCTCGAAGACGATCAGA	TTAAGCCGCAGGCTCCACTC
geneMaker00012327	GCTCGCCCTCTCCTCCATACTC	TGATCTGCTGCTTTTCTCGCTTGG
geneMaker00014156	ACTGCTGACGGAGACGAAGGAG	CGCACCCTTGAACCACCTCTTG
geneMaker00012243	AGCCACTACGATGACCACTCTCC	CAGCCCAAACCTCCCAGCAAATAG
geneMaker00006430	GGTGTCCGGTCGAAAGTTGTCTG	GAGGTGAAAAGGGTAGCAGCAAGG
geneMaker00016425	AAGAAAGCCCCGCCCAAGAAATC	ACCCGTAGTCGCCCACCAAG
geneMaker00013669	AGTCTCGTCCTCACCTGTTCTTGG	GCCCTTGTTTGACCCTCTTTCCTC
geneMaker00002773	CACCGCCACAATCTCTCCTCATTC	AGGGTCATCTTGGTCTGGGAAGC
geneMaker00006646	GATTGGCAATGTTGGCAGTGATGG	GGATCAGCAAGGTGGGCAAAGAG
geneMaker00007856	GCGGGAGGCAGAGGAGATGAG	CAGGCTTGCTCTTGCTGACACTC
geneMaker00013686	AGTCTCGTCCTCACCTGTTCTTGG	GCCCTTGTTTGACCCTCTTTCCTC
