# Supplementary figures and images for: 2,2′,4,4′-Tetrabromodiphenyl Ether (BDE-47) at Environmental Levels Influenced Photosynthesis in the Mangrove Species Kandelia obovata
Source: Toxics. 2024 Jun 25;12(7):456. doi: 10.3390/toxics12070456 (PMC11281169; doi:10.3390/toxics12070456)

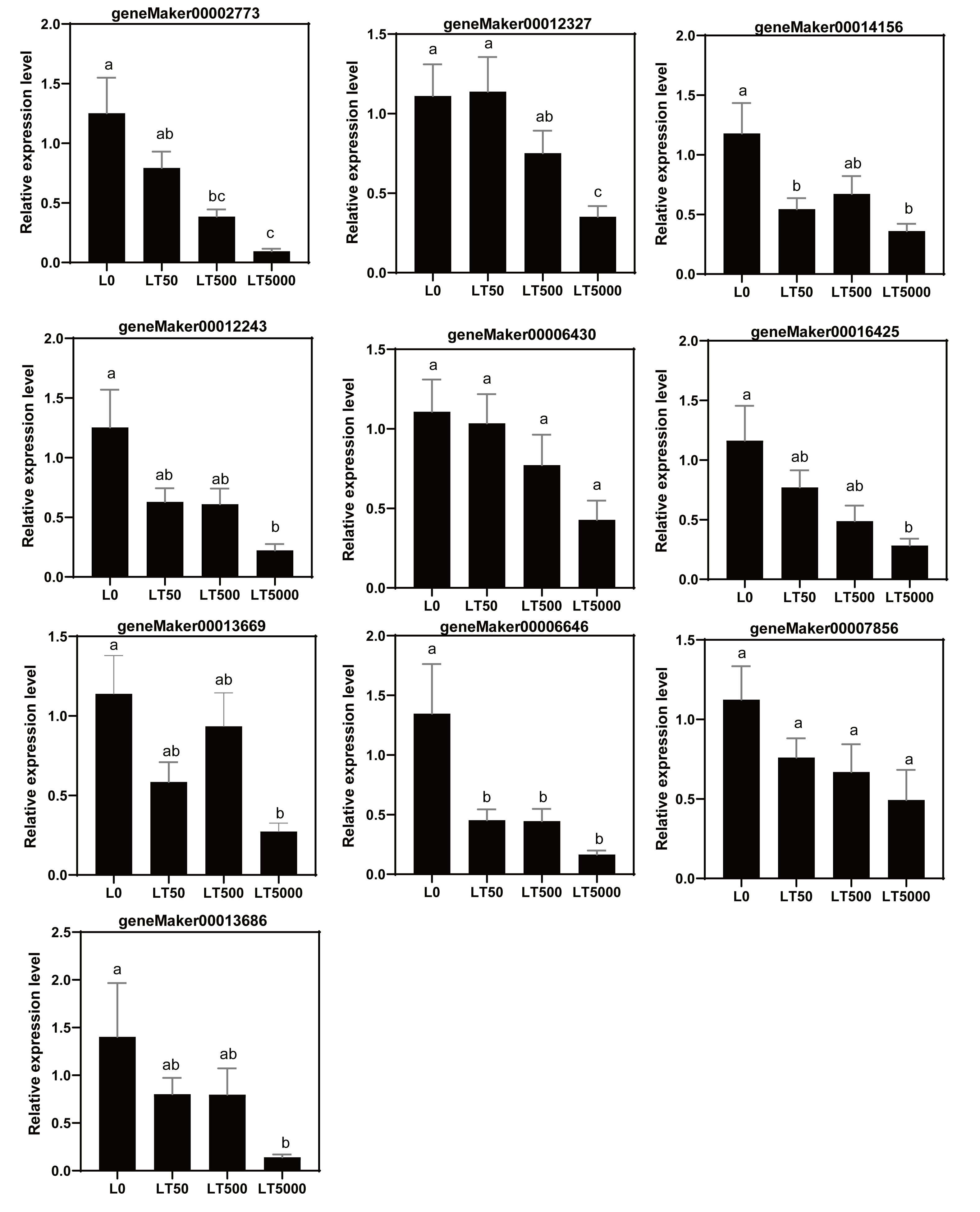

Supplement: Supplementary file 1 [file toxics-12-00456-s001.zip › Figure S1.tif]
